# Development of a lateral flow test for bed bug detection

**DOI:** 10.1038/s41598-020-70200-0

**Published:** 2020-08-07

**Authors:** Alexander Ko, Dong-Hwan Choe

**Affiliations:** 1 Division of Bayer CropScience LP, Bayer Environmental Science, 5000 Centregreen Way, Cary, NC USA; 2 Department of Entomology, University of California, Riverside, CA USA

**Keywords:** Biological techniques, Biotechnology

## Abstract

Lateral flow strip tests are a cost-effective method for detecting specific proteins in biological samples, which can be performed in the field without specialized expertise. While most recognizable in the pregnancy tests, there are many other applications for lateral flow strip technology. The pest control industry has increasingly emphasized the importance of pest monitoring to reduce unnecessary applications, focus interventions into locations with active pest infestations, and to develop records of pest infestation. Due to their cryptic behavior, the detection of bed bugs often necessitates labor-intensive, time-consuming and invasive visual inspections. A lateral flow strip test for the detection of bed bugs would represent a novel use for a well-established technology, which can enable pest control operators to rapidly confirm the presence or absence of bed bugs in a room. In the current report, we present an effort to develop and calibrate the lateral flow test devices for the detection of a bed bug specific protein. A variety of bed bug residue samples were prepared by varying several parameters: bed bug infestation level (1 bed bug/3 bed bugs), surface type (wood/fabric), feeding status (fed/unfed), and bed bug time-on-surface (1 d/7 d). Using a prototype sensor and test strip, we examined how these variables influenced the detection of the bed bug specific proteins in the sample and to what degree. We discuss how this lateral flow test device can be an effective tool to determine the presence or absence of bed bug proteins on a surface, providing highly credible evidence on bed bug infestations.

## Introduction

As haematophagous insects, bed bugs (*Cimex lectularius*, L.) feed exclusively on blood for survival, with nymphal instars requiring blood meals for development, and adult females requiring blood for reproduction^1^. Bed bug infestations can have tremendously negative effects on human health and quality of life. Aside from the bed bug bite itself, which could result in lesions, bullous eruptions, and macular spots^2^, the presence of bed bugs and the knowledge of having been bitten can induce psychological trauma, depression, anxiety, and paranoia^3^. With increasing reports of bed bug infestations throughout the world, existing evidences indicate that bed bugs have been resurging for the past 20 years^4^.

When the infestation levels are relatively low, bed bugs are notoriously difficult to spot, due to their nocturnal biology and their tendency to hide in cracks and crevices out of sight from their host^1^. Because of this cryptic lifestyle, controlling bed bug infestations is also a difficult and labor-intensive process, often necessitating the use of chemical treatments or heat treatments. Currently, most of the services available for bed bug control are curative and remedial. These reactive approaches can be less effective in multi-family or high-occupancy housing settings in which bed bugs can easily transfer from infested units to un-infested units^5,6^ through social contact^7^ and shared spaces^6^. If a pest control operator lacks access to infested rooms, the prolonged infestation can bloom out of control as the infested unit serves as a refuge for bed bugs^8^.

Proactive and early detection of bed bugs is crucial in maximizing control efforts, reducing the need for repeated visits and minimizing the likelihood of a chronic infestation^8–10^. There are several bed bug detection devices currently available in the market today. These range from inexpensive ‘passive monitors (no lure)’ such as sticky traps and pitfall traps to more elaborate and expensive ‘active monitors (with lure)’. Sticky traps are not effective in detecting bed bugs^11^ despite their widespread use (67.9% of pest control operators use sticky traps for bed bug detection^12^). In contrast, pitfall traps are effective in detecting low-level bed bug infestations^5,6,9,10,13–15^. However, both sticky trap and pitfall trap require at least two separate visits by the pest control operator (one visit for installation and a separate visit to inspect traps). Passive monitors also require regular service (cleaning and/or relubrication), as dust and debris buildup can render both sticky traps and pitfall traps ineffective^16^. While active bed bug traps with lures generally entail greater cost, the detection rate of these devices vary significantly^14,17^ and likely depends upon the design of the trap^18^ and the type of active lure being used^19^.

Bed bug detection services are also commonly offered, such as human inspection by a pest control operator and the use of canine bed bug detection teams. Like the devices, these also vary in detection accuracy and cost. Detailed visual inspections (requiring 42–90 min per apartment) by an experienced pest control operator only yield a 52% accuracy rate^6,20^, and canine bed bug sniffing dogs vary significantly in their detection rate (44%) among teams and within teams over the span of several days^15^. No device or service has yet been able to achieve high levels of bed bug detection accuracy, combined with low cost, and real-time results.

Since its first commercial application in the widely available pregnancy tests, the lateral flow strip technology has found additional applications in the detection of pathological elements for the military (bio-defense), infection/contamination detection, presence of toxic compounds in food/feed, and presence of illicit drugs^21^. By simply applying a liquid sample (containing an analyte) to a cellulose membrane (or other wicking membrane), cost effective detection of biological compounds can be achieved using complementary antibodies^21^. These gold conjugated antibodies are then bound to a complementary antibody on a region of the strip called the test line^21^. If the sample contains the protein of interest, the test line will show up with color, indicating that the protein has been bound^21^. The appearance of color at the control line ensures that a strip is functioning properly^21^. These point-of-care devices are inexpensive to manufacture and simple to use; they enable accurate and robust biological testing in areas that lack access to scientific equipment^21^.

In this paper, we describe the use of lateral flow strip technology for the detection of bed bugs. One of the major objectives of the research was to calibrate lateral flow strips for the accurate detection of bed bug residues. Several different surfaces containing bed bug residues were prepared by varying the number of bed bugs on the surface (1 vs. 3 bed bugs), the feeding status of the bed bug (fed vs. unfed), the substrate type (fabric vs. wood), and the amount of time the bed bug spent on the substrate (1 day vs. 1 week). Including no bed bug controls (clean fabric or wood surfaces without bed bug contact), a total of 18 different surfaces were sampled with special swabs. The swabs were then shipped to an external lateral flow assay laboratory where they were analyzed using a prototype sensor and test strip. The accuracy of the lateral flow strip device was determined in discriminating infested from non-infested (control) swabs.

## Results

**Figure 1:**
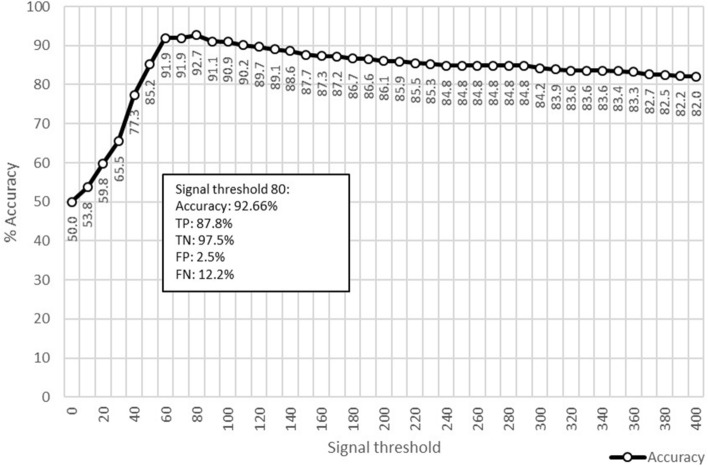
Calculation of accuracy given signal thresholds ranging from 0 to 400 (x-axis). More detailed breakdown of true positive/negative and false positive/negative is provided in the box for the two signal thresholds with the highest accuracy.

**Figure 2:**
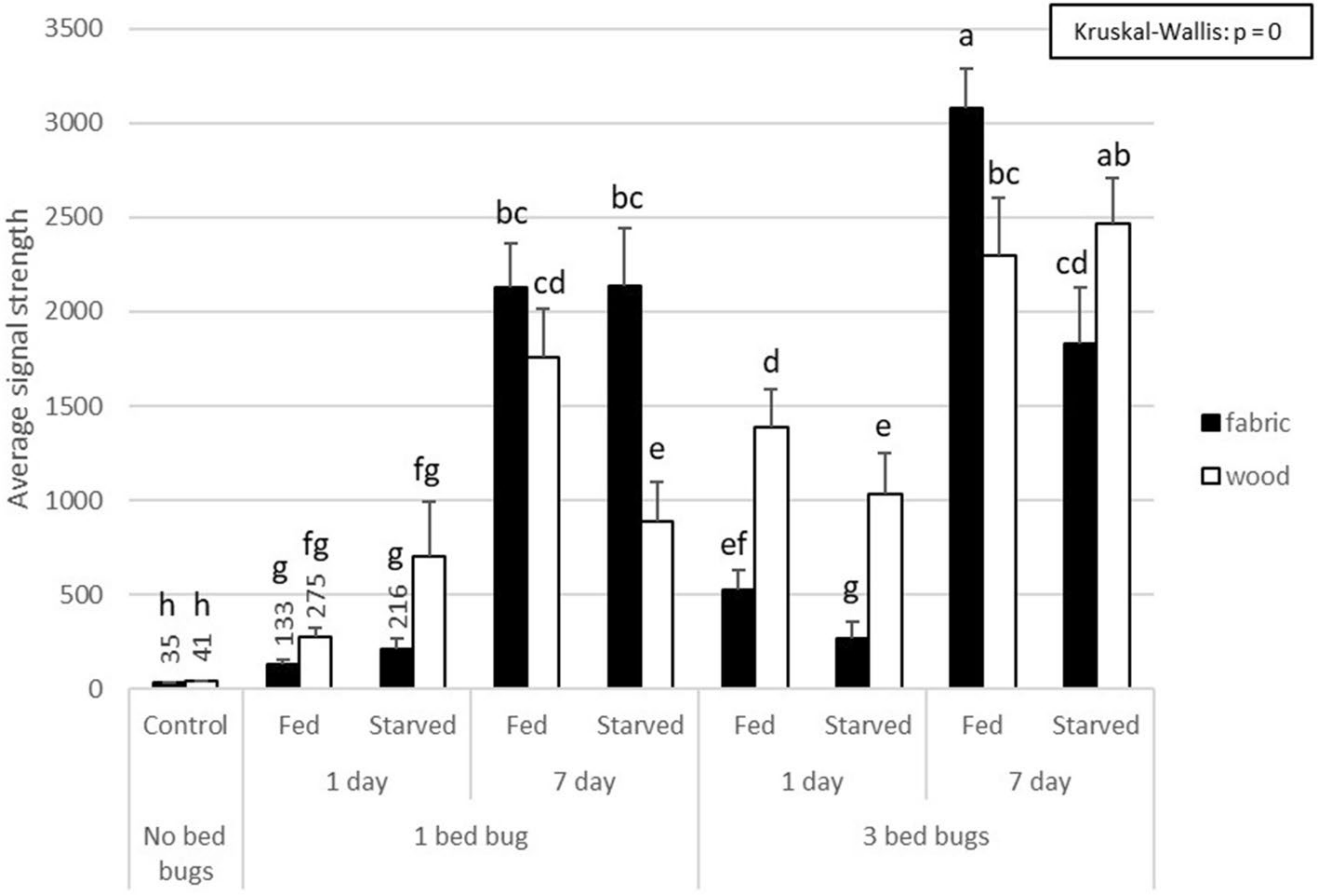
Signal differentiation among treatments (mean $\pm$ SE). Different letters indicate significant differences. Note that control samples are below the signal threshold of 60 and are significantly less than the other swab samples taken from bed bug infested surfaces.

A necessary component to understanding the theoretical maximum detection accuracy of the lateral flow strips is understanding the signal values of swabs taken from clean/control surfaces compared to signal values of swabs taken from bed bug contaminated surfaces. The value that most differentiates these control surfaces from infested surfaces is the signal value threshold. The results indicate a theoretical maximum accuracy range of 92.66% at a signal value threshold of 80 (Fig. 1). The swabs taken from control surfaces exhibited significantly lower sensor values compared to the swabs sampled from bed bug contaminated surfaces (Fig. 2). At a signal threshold of 80, accuracy was 92.66%, true positive rate was 87.81%, true negative rate was 97.5%, false positive rate was 2.5%, and false negative rate was 12.18%.

### Effect of variables on signal strength

The number of bed bugs on the surface (1 vs. 3 bed bugs) and the amount of time the bed bugs spent on the surface (1 day vs. 1 week) both had significant effects on sensor values (GLM, *p* < 0.01 and *p* < 0.001, respectively). However, substrate the bed bugs rested on (fabric vs. wood), and the feeding status of the bed bugs (fed vs. unfed) did not have a significant effect on sensor values (GLM, *p* = 0.75 and *p* = 0.19, respectively).

### Average signal strength across all treatments

Sensor values of control swabs (taken from fabric and wooden surfaces without bed bug contact) were significantly lower than those obtained from the swabs taken from infested surfaces (Kruskal–Wallis test: critical value = 243.6511, df = 17, *p* = 0) (Fig. 2).

## Discussion

In order to achieve satisfactory bed bug control, pest control operators must be able to detect low-level bed bug infestations. If low-level infestations can be efficiently detected for early intervention, the potential for bed bug introduction into adjacent areas could be also minimized. Therefore, in order to develop a truly proactive bed bug management service, pest control operators must be able to detect even low-level infestations.

We find that lateral flow strip technology is extremely accurate (92.66% @ signal threshold of 80) in detecting even low-level bed bug residue on both fabric and wooden surfaces. It is important to note that a single starved bed bug can produce enough residue to be detected after just one day (Fig. 2), demonstrating the sensitivity of the lateral flow strip technology. Control swabs of both fabric and wood substrates exhibited significantly lower signals than all the infested swabs (Fig. 2). Our finding that sensor values taken from fabric surfaces did not vary significantly from sensor values taken from wooden surfaces is a positive result (Table 1), as pest control operators can be expected to use a bed bug detection tool on a variety of surfaces; thus, sensor values and the likelihood of bed bug detection should not vary significantly among surfaces swabbed. Our findings that neither low numbers of bed bugs on the surface nor shorter times on surface (Table 1) affected our ability to detect bed bugs with the indicated accuracy, is a great boon to our confidence that lateral flow strip technology is sensitive enough to reliably detect even low-level bed bug infestations. We were surprised that the differences in sensor values between fed and starved bed bugs were not significant (Table 1), as fed bed bugs will deposit more fecal material than starved bed bugs and could therefore be expected to leave behind more contaminated material for the swab to pick up. It is possible that feces of the fed bed bugs were deposited shortly after the blood meal, but before the bed bugs were placed onto the testing surface, as Salazar et al.^22^ determined the median time between end of feeding and first defecation for *Cimex lectularius* to be 5.1 min. However, without knowing the specific antigen that is being targeted by the antibody, it is not possible at this time to know if the targeted antigen is present in bed bug feces.

**Table 1:** Generalized linear model testing the effects of bed bug density, time on surface, substrate, and feeding status of bed bugs on signal strength.

Effect	Estimate	SE	*t* value	*p*	Significance
**Generalized linear model**					
(Intercept)	2.21E-03	2.10E−04	10.535	< *2e−16*	***
Number of bed bugs	− 1.33E−04	4.45E−05	− 2.993	*0.00298*	**
Time	− 2.08E−04	2.65E−05	− 7.855	*6.35E−14*	***
Substrate	− 2.45E−05	7.82E−05	− 0.313	0.75478	
Feeding status	1.05E−04	8.03E−05	1.311	0.1908	

***p < 0.001, **p < 0.01, *p < 0.05.

### Device limitations

Because the lateral flow strip detects bed bug residue, the device might not be able to differentiate between old residue and new residue. As such, pest control operators that only employ bed bug monitoring devices (such as pitfall traps) after a bed bug treatment (such as heat treatment) may not capture the full benefit of such a device. Additional research is needed to determine the effect of heat on the analyte within bed bug residues, as typical heat treatments reach temperatures of 135–140°F for several hours^20^. Lastly, it is unknown whether the analytes within bed bug residues will be affected by external factors such as time and cleaning products, or internal insect specific factors such as bed bug strain, life stage, and sex, which might affect the utility of the device in the field. Additionally, it is currently unknown what the specific biological protein target is that the antibodies are binding to, but future research will address this.

### Potential use in the field

It is currently unknown how well the device will detect real in-field bed bug infestations, but future research will address detection accuracy of the device in the field, especially compared to existing bed bug detection methods. Because the swab is dependent on the collection of the targeted antigen on a surface, designing the ideal swabbing protocol would be necessary in order to maximize the likelihood of capturing the targeted antigen (e.g., targeting the mattress, boxspring, and other areas in which the person can be expected to remain for an extended period of time). In addition to using the device to detect the presence of bed bugs, conversely, the device and swab could also potentially be used to confirm that a surface is not contaminated with bed bugs. Furthermore, device users may use different numbers of swabs to address different questions. For instance, a user could potentially use a single swab on multiple surfaces in a room (char, couch, mattress, bedspring, headboard) to get a general sense of whether bed bug residue is detected. If no bed bug residue is detected, the evaluation can stop. If bed bug residue is detected, additional swabs can be used to pinpoint the specific item in the room responsible for the positive bed bug result.

### Technology shapes expectation

Detection of bed bugs will play a more important role in the future beyond the enhanced control of the pest. As the pace of technology increases and detection accuracy with technologically sophisticated tools also increase, we may find that our standards for pest-free environments are likewise elevated. For example, landlords, property owners^23^, and hoteliers may find that there is value in being able to certify that their structures are bed bug free. In New York, property owners are now required to provide potential renters with a bed bug infestation history for the unit that is being rented^23^. It is interesting to postulate what might happen if general consumers such as hotel guests and travelers are given the ability to detect bed bugs with a high degree of accuracy; how might bed bug detection services and expectations be shaped by such a technology in the future?

## Conclusion

Pest control operators are in need of accurate, cost-effective, and rapid bed bug detection tools. Availability of such technology will greatly enhance the ability of pest control operators to both proactively inspect areas for bed bug activity and focus treatments on areas of infestation. Logically, bed bug detection devices are unnecessary when bed bug infestations are extreme, so the ability to detect low level infestations is extremely important for proactive bed bug detection. Modern advances in lateral flow strip technology might offer pest control operators the ability to perform advanced biological analyses in a cost-effective package. In the future, as point-of-care devices continue to develop, pest control operators will have access to advanced technological tools with which they can perform sophisticated biological analyses that were once unavailable.

## Methods

### Insect

Earl strain of bed bugs maintained in laboratory were used in the current study. This strain was purchased from Sierra Research Laboratories (Modesto, CA) in 2012. The Earl strain was originally collected in Modesto, CA, in 2007. Laboratory bioassays indicated that this strain was susceptible to technical grade permethrin and deltamethrin (B. Donahue, unpublished data). Only adult male bed bugs were used in the current study.

### Treatments

We exposed one or three, fed (1 or 2 days post-feeding) or unfed (> 7 days post-feeding) bed bugs on either fabric (cotton) or wooden surfaces (bass wood panel) for either 1 or 7 days. The bed bugs were confined within a circular area (23 mm in diameter) on the surface of substrates by covering them with 12-well cell culture plates (Corning Inc., Corning, NY). After removing bed bug from the substrates (with a brief flow of CO_2_), the circular area was sampled with a swab. While gently pressing the side of swap against the substrate, a circular movement (1 rotation) was used to sample the entire area where the bed bug(s) might have contacted (see video in supplementary information). For each surface sample, two swaps were consecutively collected. These swaps were placed into a sterile plastic culture tube (15 ml) and capped. Overall, we evaluated 18 different treatment combinations varying in bed bug density (2 levels), exposure time (2 levels), feeding status (2 levels), and substrate type (2 levels), including two controls (no bed bugs). Control swabs were prepared by swabbing either the fabric or wooden surface alone. Treatments and controls were replicated 10 times.

Swab samples coded with a unique identification number and processed at Lumos Diagnostics in Carlsbad, CA (https://lumosdiagnostics.com/). Lumos Diagnostics extracted each swab into individual buffer solution (50 mM Tris–HCl + 0.1% BSA + 0.1% Tween-20 + 0.05% Azide, pH 7.6), which were then placed into the lateral flow assay device. The test was conducted in a blind fashion, as Lumos Diagnostics did not have the treatment assignments that each swab sample belonged to. After 5 min the lateral flow assay test was concluded, and sensor value was read. Higher sensor values indicate greater likelihood of the presence of bed bug residues collected on the swab. Our challenge was to identify the sensor value that most differentiates the control swabs (noise) from the infested swabs (signal); i.e., the signal threshold. Identifying this signal threshold would also enable the calculation of accuracy of the device.

### Lateral flow strip device

The lateral flow strip device uses a pair of optical sensors with an illumination source that is positioned between the test line and the control line on the cellulose lateral flow assay strip. These optical sensors calculate the reflectance of the light from the white cellulose strip. Decreased reflectance is correlated with increased sensor values from the optical sensors, which indicate that the protein/colloidal gold antibody complex have successfully attached to the region of the strip being analyzed; i.e., either the test line or control line.

### Sensor values

Sensor values are calculated with the following calculation:

$$ {\text{Sensor}}\;{\text{value }} = \, \left[ {\left( {{\text{Ri}}/{\text{R}}} \right) \, {-} \, \left( {{\text{Fi}}/{\text{F}}} \right)} \right] \, *{\text{ SF}} $$

Ri = Initial reading from rear sensor (control line), R = Current reading from rear sensor (control line), Fi = Initial reading from front sensor (test line), F = Current reading from front sensor (test line), SF = scaling factor where the scaling factor is a large positive integer and is used to avoid values less than one in the calculated result.

Thus, swabs sampled from bed bug infested substrates are expected to have high sensor values as the upstream test line captures more protein bound gold-conjugated antibodies, decreasing the reflectance of light back into the optical sensor relative to the downstream control line (Patent Publication #: US 2017/0,115,301).

### Calculation of accuracy

Accuracy was calculated using the following formula:

$$ {\text{Accuracy}} = \left( {{\text{TP}} + {\text{TN}}} \right)/\left( {{\text{TP}} + {\text{TN}} + {\text{FP}} + {\text{FN}}} \right) $$

TP = True positive (instances in which the test predicts positive and actual value is positive), TN = True negative (instances in which the test predicts negative and actual value is negative), FP = False positive (instances in which the test predicts positive and actual value is negative), FN = False negative (instances in which the test predicts negative and actual value is positive).

Figure 1 was created by calculating the total accuracy of the swabs given a theoretical signal threshold, ranging from 0 to 400. For example, because signals from the electronic reader cannot be negative, a signal threshold of 0 would result in a 0 TN rate and a 0 FP rate. As the signal threshold increases in increments of 10, the accuracy is calculated until a theoretical maximum accuracy rate emerges.

### Data analysis

Data was analyzed using the R statistical program (version 3.6.2) and Microsoft Excel. P-values less than or equal to 0.05 were regarded as significant. For data shown in Fig. 2, data failed to satisfy parametric assumptions of skewness, kurtosis, and heteroscedasticity, so a non-parametric Kruskal–Wallis test was performed using the Agricolae package (version 1.3–1) in R. For the analysis of the significance of the treatments (number of bed bugs on surface, bed bug time on surface, substrate type, feeding status) in affecting signal strength, we used a GLM (Generalized Linear Model) analysis (base stats package 3.6.2) in R (R foundation for Statistical Computing; Vienna, Austria).
